# The antiosteoporotic effect of oxymatrine compared to testosterone in orchiectomized rats

**DOI:** 10.1186/s13018-024-05344-0

**Published:** 2025-01-09

**Authors:** Anwaar M. Shaban, Eman A. Ali, Sara G. Tayel, Sara Kamal Rizk, Dalia F. El Agamy

**Affiliations:** 1 Medical Physiology Department, Faculty of Medicine, Menoufia University, Menoufia, Egypt; 2 Clinical Pharmacology Department, Faculty of Medicine, Menoufia University, Menoufia, Egypt; 3 Anatomy and Embryology Department, Faculty of Medicine, Menoufia University, Menoufia, Egypt; 4 Medical Biochemistry and Molecular Biology Department, Faculty of Medicine, Menoufia University, Menoufia, Egypt; 5 Clinical Pharmacology Department, Faculty of Medicine, Menoufia National University, Menoufia, Egypt; 6 Anatomy and Embryology Department, Faculty of Medicine, Menoufia National University, Menoufia, Egypt; 7 Medical Physiology Department, Faculty of Medicine, Menoufia National University, Menoufia, Egypt

**Keywords:** Orchiectomy, Osteoporosis, Oxymatrine, Testosterone

## Abstract

**Background:**

Castration of adult male rats led to the development of osteoporosis. Oxidative stress and inflammatory factors have been identified as potential causative factors. Notably, oxymatrine (OMT) possesses potent anti-inflammatory and antioxidant activities. This study aims to elucidate the antiosteoporotic effects of OMT compared to testosterone in an orchiectomized (ORX) rat model of osteoporosis.

**Methods:**

A total of 60 Wistar male rats were divided into the following groups: control (CTRL), surgery + no orchiectomy (SHAM), ORX, ORX + testosterone, and ORX + OMT. Urinary deoxypyridinoline (DPD), calcium (Ca), and phosphorus (P), as well as serum testosterone, parathormone (PTH), alkaline phosphatase (ALP), osteocalcin, N-telopeptide of type I collagen (NTX I), tartrate resistance acid phosphatase (TRAP), and total Ca and P levels were evaluated. Bone was assessed for malondialdehyde (MDA), reduced glutathione (GSH), interleukin 6 (IL-6), Kelch-like ECH-associated protein 1 (Keap1), nuclear factor erythroid 2-related factor 2 (Nrf2), heme oxygenase 1 (HO-1) expression, and receptor activator of nuclear factor κB ligand/ osteoprotegerin (RANKL/OPG) ratio. Bone dual-energy X-ray absorptiometry (DEXA) scan and histological and immunohistochemical studies were performed.

**Results:**

Testosterone or OMT treatment ameliorated the reduced bone mineral density (BMD) and bone mineral content (BMC) in the DEXA scan and the changes in PTH and Ca levels. Compared to the ORX group, bone formation, and turnover markers were also significantly reversed in the treatment groups. Treatment with testosterone or OMT significantly reduced bone MDA, IL-6, Keap1, RANKL, and RANKL/OPG ratio, and significantly elevated bone GSH, Nrf2, and HO-1. Moreover, testosterone or OMT treatment has restored cortical bone thickness and osteocyte number and reduced bone levels of TNF-α in ORX rats. Consequently, treatment with either testosterone or OMT exhibited nearly equal therapeutic efficacy; however, neither of them could normalize the measured parameters.

**Conclusion:**

OMT treatment showed equal efficacy compared to testosterone in ameliorating osteoporosis in ORX rats, possibly by improving some inflammatory and oxidative stress parameters.

## Introduction

Osteoporosis, bone mass loss due to aging or disease, is a significant health concern among humans [1, 2]. When bone loss exceeds a specific limit, it raises the possibility of bone fracture. Hypogonadism predisposes individuals to osteopenia. Unlike female postmenopausal osteoporosis, testosterone deficiency in men has not received much attention. This may be because significant hypogonadism is generally less common in males than in females, leading to it being overlooked in discussions about health concerns [3].

Testosterone levels in males between the ages of 30 and 90 decrease progressively by 1% each year. Several diseases can impact testosterone levels, including diabetes, obesity, and endocrine therapies such as androgen deprivation treatment for prostate cancer [4]. Androgens play a crucial role in the musculoskeletal, endocrine, and reproductive systems. Testosterone is converted in the cytoplasm of target cells into a more potent form called dihydrotestosterone by the enzyme 5α-reductase, which initiates androgenic activity. Additionally, testosterone is converted to estradiol by aromatase, which plays a crucial role in bone metabolism. Male patients with aromatase deficiency experience a significant reduction in bone mineral density (BMD) in both trabecular and cortical bone. Estradiol deficiency increases inflammatory cytokines, tumor growth factor-β production, interleukin (IL)-1, IL-6, IL-7, nuclear factor-κB (NF-κB), and tumor necrosis factor-α (TNF- α) reducing osteoblast proliferation and activity while increasing osteoclastic activity and the expression of RANKL-mediated osteoclastogenesis, resulting in BMD loss. Testosterone can enhance osteoblasts’ differentiation and bone formation by directly triggering osteoblasts’ androgen receptors. Testosterone plays a vital role in bone metabolism by positively regulating several growth-promoting agents and cytokines. These include insulin-like growth factor-1 (IGF-1), IGF-binding protein, and transforming growth factor-β within osteoblasts. These factors promote the differentiation and proliferation of chondrocytes and osteoblasts while suppressing the apoptosis (cell death) of chondrocytes, ultimately leading to enhanced bone formation. Additionally, testosterone reduces the activity of (IL-6), which activates osteoclasts and promotes bone resorption. Conversely, low testosterone levels reduce BMD by activating IL-6. Furthermore, testosterone deficiency enhances the activation of receptor activator of nuclear factor kappa-B ligand (RANKL) production from osteoblasts, contributing to the activation and differentiation of osteoclasts, promoting bone resorption, and reducing BMD [5].

In today’s aging society, testosterone deficiency is considered a significant public health risk that negatively impacts the lives of older men. Testosterone deficiency can lead to erectile dysfunction, lack of energy or tiredness, depression, anxiety, BMD decline, reduced mass and strength of muscle, visceral obesity, and hair loss. Additionally, severe symptomatic androgen deprivation has been described in hormone ablation therapy in prostate cancer treatment, and testosterone should be substituted to prevent the osteoporotic side effects [6]. Males with osteoporosis are four times more likely than females to suffer from femoral neck fractures. Therefore, osteoporosis prevention is crucial for preserving daily activities and quality of life [5].

A negative correlation exists between testosterone levels and the production of reactive oxygen species (ROS) as well as proinflammatory markers like C-reactive protein and TNF-α. Moreover, testosterone levels correlate positively with mitochondrial membrane potential, superoxide dismutase, and glutathione reductase, indicating that reduced testosterone in men is linked to altered mitochondrial function and oxidative stress [7, 8]. Oxidative stress and inflammation, significantly contribute to osteoporosis by halting osteoblast differentiation, promoting osteoclast activity, increasing apoptotic osteocytes, and upregulating RANKL expression and the RANKL/osteoprotegerin (OPG) ratio [9]. The Kelch-like ECH-associated protein 1 (KEAP1)- nuclear factor erythroid 2-related factor 2 (Nrf2) system is a cellular defense mechanism that helps remove ROS and combat oxidative stress. Typically, Nrf2 is linked to actin-binding Keap1, creating the Nrf2-Keap1 complex. This complex blocks the entry of nrf2 into the nucleolus and leads to its breakdown through proteasomal degradation. However, when exposed to oxidative stress and H2O2, Nrf2 and Keap1 separate and move into the nucleolus to enhance the expression of enzymes [10]. The Keap1-Nrf2 pathway controls the expression of various cytoprotective genes, including heme oxygenase-1 (HO-1) and its products. These components help protect against oxidative damage, regulate apoptosis, modulate inflammation, and contribute to angiogenesis [11].

Oxymatrine (OMT) is a phytochemical with a broad spectrum of pharmacological properties, such as anti-inflammatory, antioxidant, and anti-apoptotic activities. It is an alkaloid separated from the customary Chinese plant sophora roots. Animal investigations reported potent therapeutic effects on liver injury, renal injury, rheumatoid arthritis, heart failure, and mastitis by inhibiting the release of various inflammatory cytokines and influencing various signal pathways. These pathways include decreasing the activation of the NF-κB pathway, reversing the oxidative stress by upregulating the Nrf2/HO-1 signal pathway, protecting the damaged organs and tissues by reducing apoptosis through increasing Bcl-2 and decreasing Bax and cleaved-caspase-3, and upregulating the PI3K/Akt/GSK-3β pathway as reported by Lan et al. [12, 13]. Interestingly, the efficacy of OMT might promise a therapeutic effect on osteoporosis in orchiectomized (ORX) rats.

This study’s objective is to clarify the antiosteoporotic impact of OMT on osteoporosis in ORX rats compared to testosterone and to examine the possible associated anti-inflammatory and antioxidative actions.

## Materials and methods

### Animals

A total of 60 age-matched adult Wister albino male rats weighing between 300 and 380 g, aged 10–11 weeks, were used in this experiment. During the study period, rats were kept in fully ventilated cages (2/cage) at room temperature, humidity, and a light/dark cycle. Before the study began, rats were given 10 days to acclimate with free access to water and a standard, pelleted commercial diet. The study adhered to AARIVE guidelines and the Guide for the Care and Use of Laboratory Animals, approved by the ethical committee at the Faculty of Medicine, Menoufia University (code: 7/2024BIO2).

### Sample size estimation

The sample size for this experimental investigation was 60 (12 rats allocated to each group), as guided by a prior study conducted by Böker et al. [14]. This sample size calculation was achieved with an 80% power at a significance level of 0.05 using G*Power software (version 3.1.9.2; Germany).

### Experimental design

The rats were randomly divided into five groups (12/group). The CTRL group received distilled water (DW) via oral gavage, while the SHAM group was exposed to all steps of orchidectomy except the removal of the testicles. The ORX group was exposed to ORX and received DW via oral gavage [15]. The ORX + testosterone group was exposed to ORX surgery and then, testosterone undecanoate was started after one month of the surgery. A low dose of testosterone hormone (testosterone undecanoate, 8 mg/kg) with the potential for treating androgen-deficient osteoporosis and helping reduce the side effects of testosterone therapy was used, as reported by previous studies [16–18]. Testosterone was purchased from TCI- UK Ltd.- (UK). Then, it was diluted in sesame oil, and a low dose (8 mg/mL) was injected via IM. route every day for six weeks [16–19]. Moreover, the ORX + OMT group was subjected to ORX surgery. After one month of ORX, animals received intragastric OMT of > 98% purity (Sigma Aldrich, MO, USA) for six weeks. The low dose of OMT (50 mg/kg dissolved in DW) was chosen based on previous in vivo studies that demonstrated the antioxidant and anti-inflammatory effects of OMT [20–22].

### Method of orchidectomy

All used surgical instruments, hard surfaces, and cages were sterilized with 70% ethanol prior to use. Animals’ weight was estimated prior to anesthesia. Thiopental sodium was intraperitoneally injected (0.5 mg/kg) to induce anesthesia [23]. Animals were then placed under a suitable heating pad to prevent heat loss. The fur on the ventral side of the scrotum was shaved bilaterally to expose the skin, which was then cleaned with 70% ethanol. A single incision (1 cm deep incision penetrating the skin) was made on the shaved side of the scrotum using a sterile scalpel. We made an incision in the cremaster muscles to access the testicular fat pad, which was located and gently pulled through the incision using sterile blunt forceps. The testicular contents were carefully exposed using sterile blunt forceps by gently releasing the testicular fat pad. Following this, the epididymis, vas deferens, and testicular vasculature were meticulously isolated with sterile forceps. To prevent hemorrhage, the vas deferens and blood vessels were ligated prior to testicular excision. The testis was removed using scissors, and the testicular sac was repositioned. The contralateral testis was removed using the same procedure. Wound closure was achieved with sterile sutures [24].

### Postoperative care

Animals were housed individually in sterilized cages and closely observed for approximately 2–4 h until they fully recovered from anesthesia. Then, a local antibiotic, fucidin cream (Leo Pharma, Egypt), was applied twice daily to the incision site for five days.

### Urine collection

One day before the study’s end, rats were allocated to metabolic cages. Then, 24-h urine samples were gathered, centrifuged at 450 g for 5 min, and preserved at − 20°C.

### Blood sample collection

After collecting urine samples, the rats were deprived of food overnight. Subsequently, retro-orbital blood samples (2 mL) were obtained into sterile plain tubes, allowed to clot for 30 min at ambient temperature, and centrifuged at 2000 revolutions per minute at 4 °C for 15 min. The resulting serum was preserved at − 80 °C for subsequent analysis.

### Bone processing

Rats were euthanized by cervical dislocation. Femora and tibiae were removed and dissected free of soft tissue. Left femora and tibiae were preserved in 70% alcohol for dual-energy X-ray absorptiometry (DEXA) scan. The right tibiae were removed, frozen, and dipped in liquid nitrogen; they were then turned into powder using a porcelain mortar and pestle. Half of the bone powder was homogenized in phosphate-buffered saline (PBS) (100 mM, pH 7.4) and centrifuged for 10 min at 10,000 g at 4 °C. The supernatant was kept at − 80 °C for measuring malondialdehyde (MDA) and reduced glutathione (GSH) levels, while the other half of the powder was used for RT-PCR analysis of IL-6, Keap1, Nrf2, HO-1, OPG, and RANKL genes. Subsequently, the collected right femora were stored in 10% formalin and used for immunohistopathological examination.

### Bone DEXA scan

The GE Medical Systems Lunar DEXA (LU43618EN, Madison, WI, USA) apparatus was utilized to evaluate BMD and BMC in the left femora and tibiae, employing the GE enCORE 2007 software designed for assessing bone density in small animals, as detailed by Xu et al. [25]. The exposure factors were set at 76 kV voltage, 0.150 mA current, and 2.00 µGy dose over 1.75 min time. The proximal ends of the femora and tibiae were chosen as the region of interest (ROI) because of the richness of cancellous bone [26].

### Estimation of urinary calcium, phosphorus, and deoxypyridinoline

The levels of deoxypyridinoline (DPD) cross-links in urine were quantified using the ELISA method with the Metra® DPD Quidel Kit (NC9954742, San Diego, CA, USA). An ELISA microplate reader from Bio-Rad (Japan) was employed in the process. The DPD measurements were adjusted based on the urinary creatinine concentration. Both urinary calcium (Ca) and phosphorus (P) levels were assessed in 24-h urine samples using a colorimetric method and Biodiagnostic kits (Cat. No. CA 12 10 and Cat No. PH 17 10, respectively), Giza, Egypt as previously described [27].

### Serum biochemical analysis

ELISA kits were provided to detect serum testosterone (ab285350, Abcam, Cambridge, UK), parathormone (PTH) (abx256366, Abbexa BV, Leiden, NL), alkaline phosphatase (ALP) (ab83369, Abcam, Cambridge, UK), osteocalcin (E-EL-R0243, Elabscience, Houston, TX , USA), N-telopeptide of type I collagen (NTX I) (E-EL-R0276, Elabscience, Houston, TX, USA), and tartrate-resistant acid phosphatase (TRAP) (E-EL-R0939, Elabscience, Houston, TX, USA). Total Ca (CA 12 10) and P (PH 17 10) provided by Biodiagnostic, Giza, Egypt, were detected by the calorimetric method as previously described [27].

### Detection of bone oxidative stress markers

Bone levels of MDA (MD25 29) and reduced glutathione (GSH) (GR 25 11) (Biodiagnostic Company, Giza, Egypt) were measured colorimetrically [28, 29].

### Real-time PCR detection of IL-6, Keap1, Nrf2, and HO-1 expression and RANKL/OPG ratio

Following the manufacturer’s guidelines for the initial PCR step, RNA extraction from the right tibia was performed using TRIzol reagent (Invitrogen, Carlsbad, CA, USA). Subsequently, cDNA synthesis from the reverse transcribed RNA was conducted, followed by quantitative PCR done in a 50μL reaction volume utilizing the Ready-Mix PCR Reaction Mix kit (iScriptTM One-Step RT-PCR Kit with SYBR® Green, Bio-Rad, USA).

The thermal cycling parameters were as follows: an initial incubation at 50 °C for 10 min, followed by denaturation at 95 °C for 5 min, then 40 cycles of denaturation at 95 °C for 10 s, annealing at 55 °C for 30 s, and extension at 55 °C for 1 min, using the Rotor Gene Q real-time PCR system (Qiagen- South Korea). Data analysis was conducted using the Applied Biosystems 7500 software, version 2.0.1. The primer sequences utilized for quantifying OPG, RANKL, Nrf2, IL-6, Keap1, and HO-1, mRNA levels are detailed in Table 1. For data analysis, we employed the comparative Ct (2-ΔΔCT) technique, with β-actin being the internal control gene for normalization. Finally, a melting curve assessment was performed to verify amplification specificity and primer dimers’ absence.

**Table 1:** IL-6, Keap1, Nrf2, HO-1, OPG, and RANKL primer sequences

	Forward	Reverse
OPG	CACAAATTGCAGTGTCTTTGGTC	TCTGCGTTTACTTTGGTGCCA
RANKL	CCCATAAAGTGAGTCTGTCC	CAATACTTGGTGCTTCCTCC
IL6	GGCACTGGCAGAAAACAACC	GCAAGTCTCCTCATTGAATCC
KEAP1	GGACGGCAACACTGATTC	TCGTCTCGATCTGGCTCATA
Nrf2	GGTTGCCCACATTCCCAAATC	CAAGTGACTGAAACGTAGCCG
HO-1	CCATAGGCTCCTTCCTCCTTTC	GGCCTTCTTTCTAGAGAGGGAATT
β–Actin	GTGACATCCACACCCAGAGG	ACAGGATGTCAAAACTGCCC

### Histological study

Specimens from the right femora were dissected and excised, then fixed in a 10% neutral formalin solution for 48 h. Then, decalcification with ethylenediaminetetraacetic acid (EDTA) was done for about 10 days. Subsequently, the specimens were processed to prepare 5–7 µm thick paraffin blocks and then for hematoxylin and eosin (H&E) staining [30].

### Immunohistochemical study

Femoral sections were subjected to deparaffinization using freshly prepared xylene, followed by rehydration through a chain of decreasing alcohol concentrations. Subsequently, endogenous peroxidase activity was inhibited by immersing tissue sections in 3% H2O2 for 20 min. An antigen retrieval process using microwave irradiation was conducted. Then, the sections were treated with a primary TNF-α antibody (rabbit polyclonal antibody AMC3012, Thermo Fisher Scientific, USA), followed by the addition of a biotinylated goat-polyvalent secondary antibody. Streptavidin peroxidase was applied to the sections, and the DAB substrate chromogen (3,3′-diaminobenzidine tetrahydrochloride) was added. Finally, the slides were counterstained with H&E according to standard protocols [31].

### Morphometric study

Morphometric analysis of osteocyte count (× 200 magnification), cortical bone thickness (× 200 magnification), and the proportion of TNF-α immunoreactive area (× 400 magnification) was conducted using Image J 1.47 v software. Then, 10 non-overlapping fields were randomly captured for each specimen, without overlap, utilizing a Leica DML B2/11888111 microscope outfitted with a Leica DFC450 camera for every parameter.

### Statistical analysis

The data were parametric according to the Shapiro–Wilk test and were analyzed using one-way ANOVA to detect significance among groups. Bonferroni’s test was employed for multiple comparisons. The data were expressed as mean and standard deviation (SD). Statistics were applied using GraphPad Prism software (GraphPad ver. 9.3.1, USA). The *p* < 0.05 was considered statistically significant.

## Results

### The effect of testosterone or OMT on BMD and BMC

**Fig. 1:**
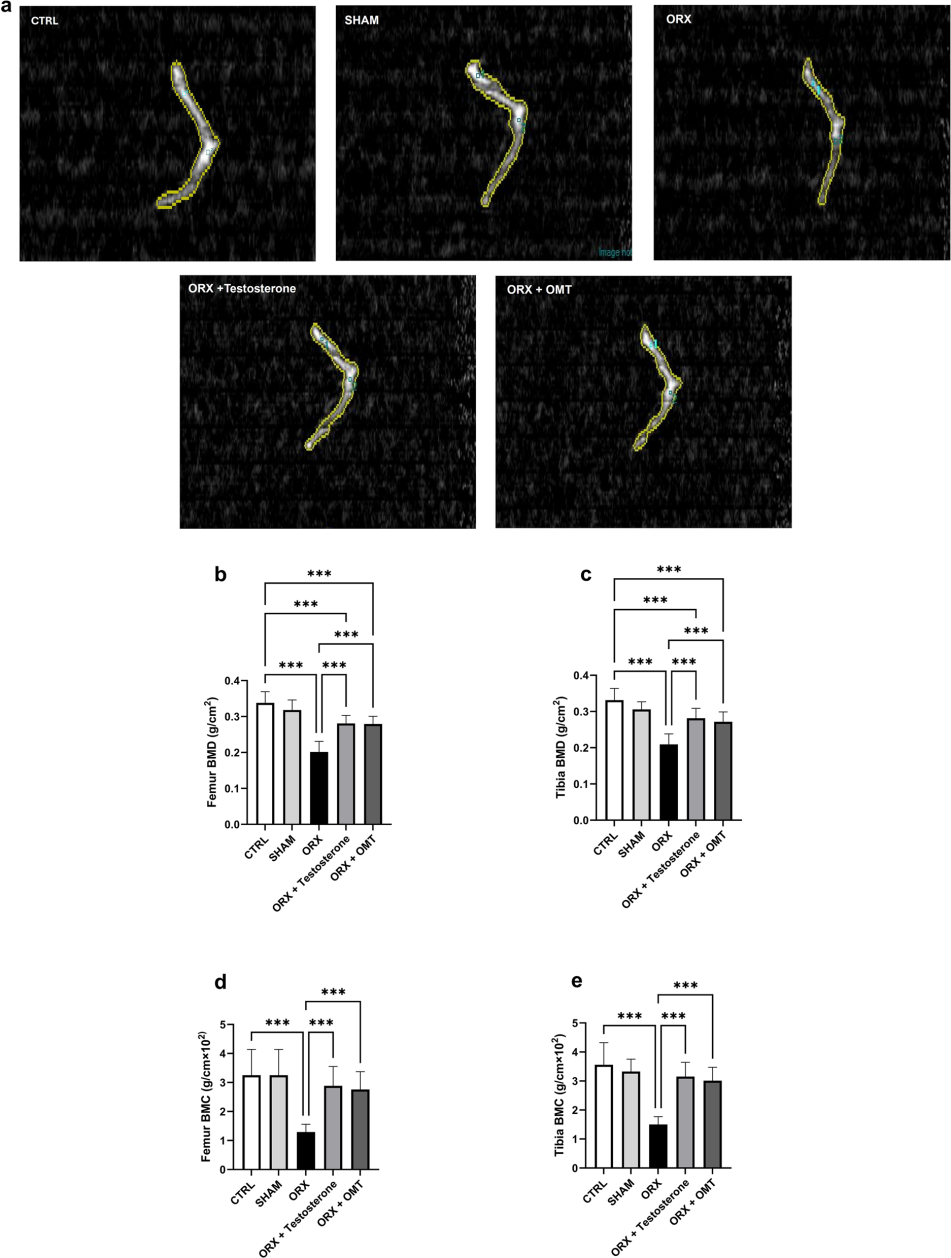
Testosterone or OMT alleviated BMD and BMC in ORX rats. **a** Representative DEXA scan images of the study group; **a** femur BMD; **c** tibia BMD; **d** femur BMC; **e** tibia BMC. Bar graphs were presented as mean ± SD (n = 12). Data analysis was conducted using one-way ANOVA and Bonferroni’s test for multiple comparisons. ^***^p < 0.001. BMD, bone mineral density; BMC, bone mineral content; ORX, orchiectomized; OMT, oxymatrine

Osteoporosis was evidenced by performing a DEXA scan, revealing that the BMD in the femora and tibiae of the ORX group declined compared to the CTRL rats (*p* < 0.001). The femora and tibiae exhibited enhanced BMD with testosterone or OMT treatment relative to the femora and tibiae of the untreated ORX rats (*p* < 0.001). The BMD in the OMT-treated group was insignificant compared to the testosterone-treated group (*p* > 0.99). However, compared to CTRL rats neither testosterone nor OMT treatment was able to normalize BMD (*p* < 0.001) (Fig. 1a–c).

Similarly, BMC was significantly reduced in the femora and tibiae of the ORX rats compared to the CTRL rats (*p* < 0.001)**.** In contrast, both testosterone and OMT treatments led to a significant increase in BMC compared to the untreated ORX controls (*p* < 0.001). No significant difference was observed in BMC between the OMT and testosterone treatment groups (*p* > 0.99). Furthermore, neither treatment group achieved the CTRL group’s levels of BMC (BMC femora: *p* > 0.99, *p* = 0.65, respectively; BMC tibia: *p* = 0.38, *p* = 0.37, respectively) (Fig. 1a, d and e).

### The effect of testosterone or OMT on serum testosterone, PTH, Ca, and P levels

**Fig. 2:**
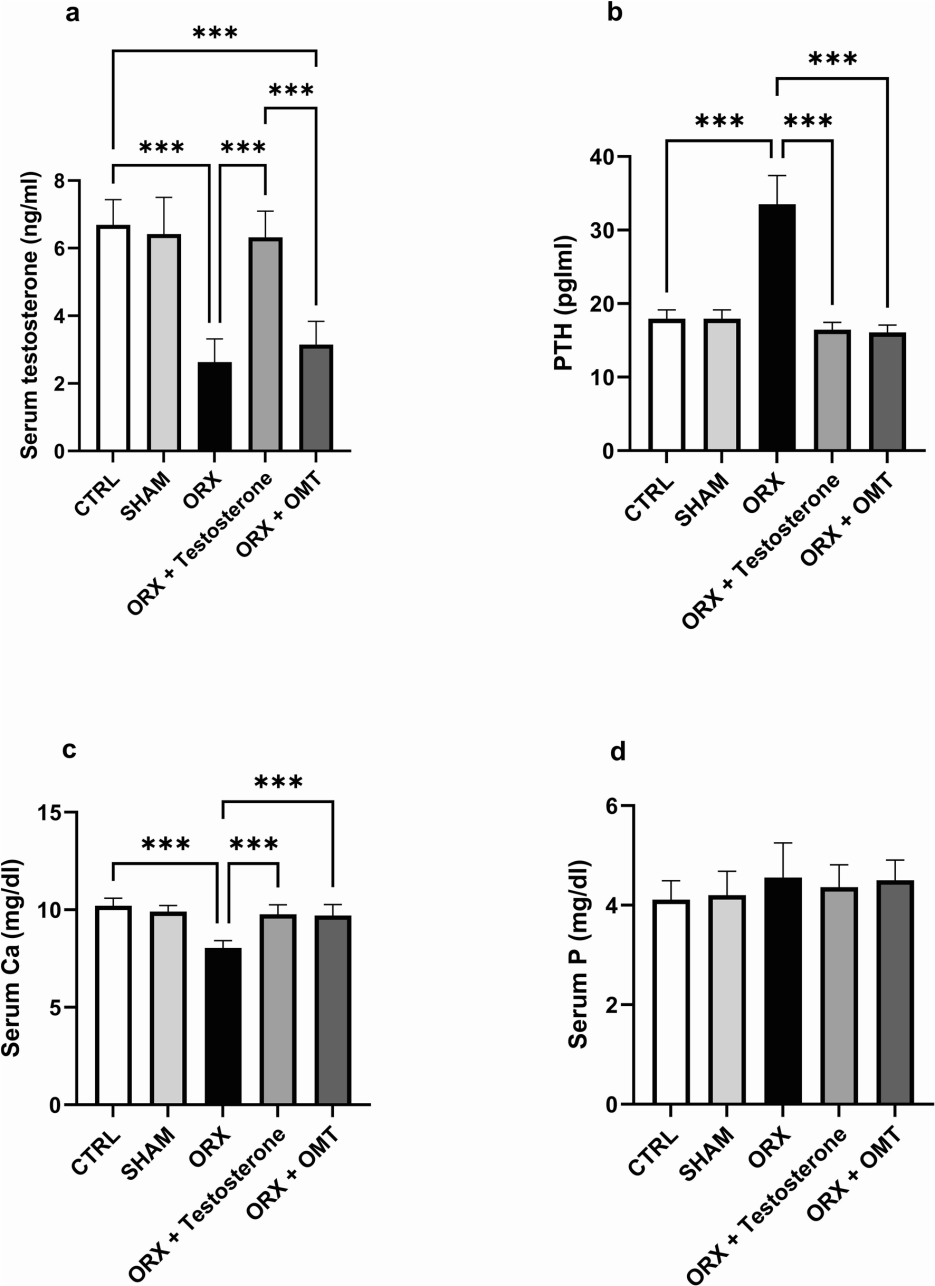
Testosterone or OMT alleviated serum testosterone, PTH, Ca, and P levels in ORX rats. **a** Serum testosterone; **b** serum PTH; **c** serum Ca; **d** serum P; **e** urine Ca; **f** urine P. Bar graphs were presented as mean ± SD (n = 12). Data analysis was conducted using one-way ANOVA and Bonferroni’s test for multiple comparisons. ^**^p < 0.01 and ^***^p < 0.001. ORX, orchiectomized; OMT, oxymatrine; PTH, parathormone; Ca, calcium; P, phosphorus

Figure 2a–d illustrated the absence of significance between the CTRL and SHAM groups regarding serum testosterone (*p* > 0.99), PTH (*p* > 0.99), Ca (p = 0.7), and P (*p* > 0.99) levels. In contrast to the CTRL group, the ORX group exhibited a significant reduction (p < 0.001) in serum testosterone levels and an elevation in PTH levels (p < 0.001). Conversely, testosterone treatment markedly elevated testosterone levels (p < 0.001) compared to those in the ORX group. Unlike testosterone, OMT failed to affect serum testosterone levels (*p* = 0.87).

Testosterone or OMT treatment reduced PTH hormone levels compared to the ORX group (p < 0.001). Both treatment groups exhibited nearly identical reductions in PTH hormone levels (*p* > 0.99). Regarding serum mineral levels, Ca levels declined (p < 0.001), while P levels showed nonsignificant change between the ORX and the CTRL groups (*p* > 0.99). Testosterone or OMT-treated groups displayed higher serum Ca levels than the ORX group (p < 0.001). No distinction was observed in Ca levels (*p* > 0.99) between the groups treated with testosterone or OMT (*p* > 0.99). Moreover, serum PTH, Ca, and P levels in the testosterone or OMT groups were almost the same as those in the CTRL group (*p* = 0.51 and *p* = 0.19, *p* = 0.13 and *p* = 0.06, *p* > 099 and *p* = 0.40, respectively). In terms of serum testosterone, no variation was noticed between the CTRL rats and the testosterone-treated rats (*p* > 0.99). However, the testosterone levels were lower in the OMT-treated rats than those in the controls (p < 0.001).

### Testosterone or OMT effects on serum ALP, osteocalcin, NTXI, and TRAP levels

**Fig. 3:**
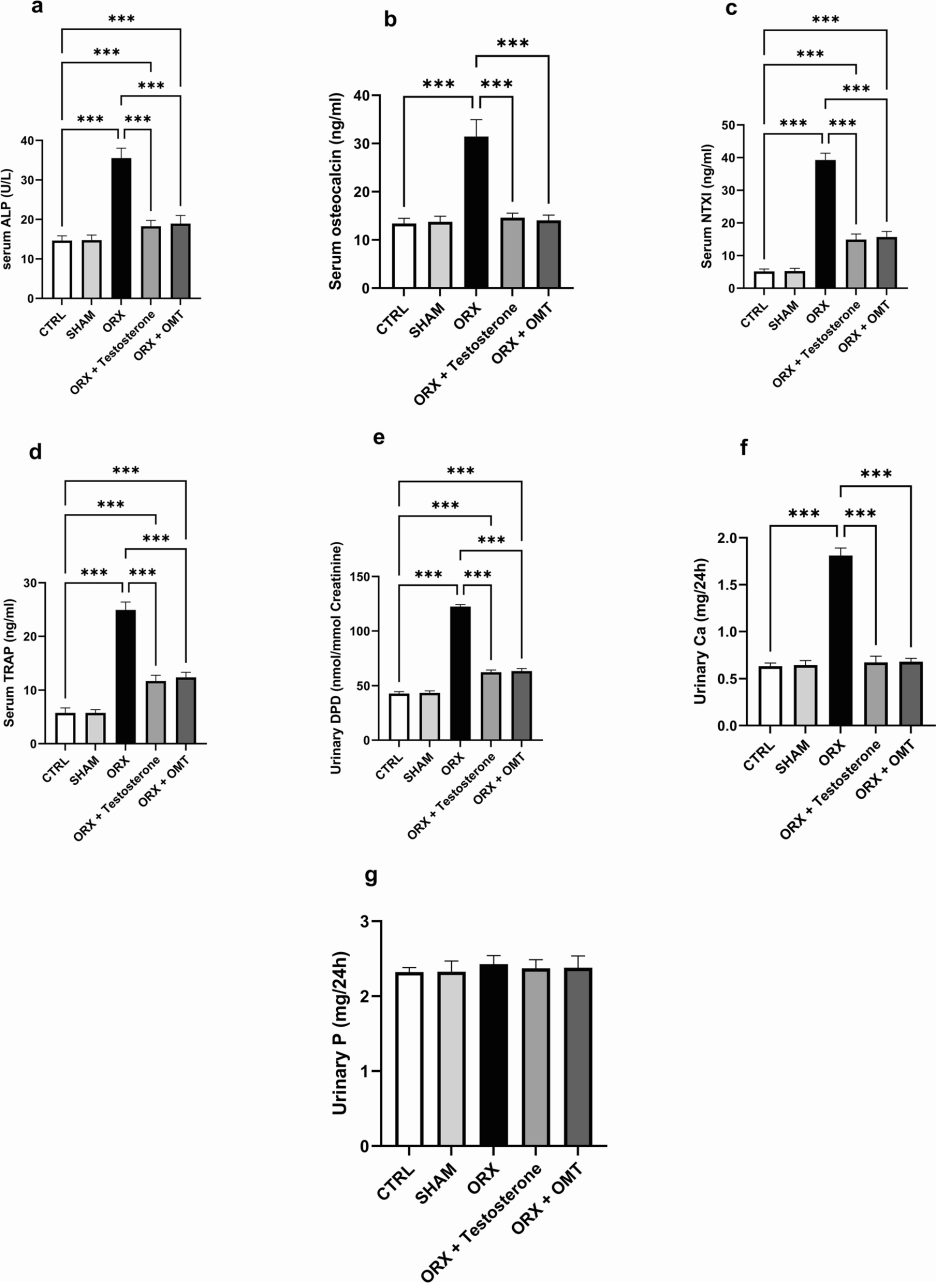
Testosterone or OMT alleviated serum ALP, osteocalcin, NTXI, TRAP, urinary DPD, Ca, and P levels in ORX rats. **a** Serum ALP; **b** serum osteocalcin; **c** serum NTXI; **d** serum TRAP; **e** urinary DPD; **f** urinary Ca; **g** urinary P. Bar graphs were presented as mean ± SD (n = 12). Data analysis was conducted using one-way ANOVA and Bonferroni’s test for multiple comparisons. ^***^p < 0.001. ORX, orchiectomized; OMT, oxymatrine; ALP, alkaline phosphatase; NTXI, N-telopeptide of type I collagen; TRAP, tartrate resistance acid phosphatase; DPD, deoxypyridinoline; Ca, calcium; P, phosphorus

Serum levels of ALP, osteocalcin, NTXI, and TRAP were comparable between the SHAM-operated and CTRL groups (*p* > 0.99). In contrast, ORX rats exhibited a significant elevation in these serum markers compared to the CTRL group (*p* < 0.001). Testosterone- and OMT-treated rats displayed a notable decline in the above-mentioned markers in relation to the ORX rats (*p* < 0.001). Changes in serum levels of ALP, osteocalcin, NTXI, and TRAP of the OMT-treated group were not statistically significant compared to the testosterone-treated group levels (*p* > 0.99, *p* > 0.99, *p* = 0.081, and *p* > 0.99, respectively). Conversely, a single treatment with either therapy failed to achieve normal CTRL values of ALP, NTXI, and TRAP levels (*p* < 0.001). In discrepancy, normal values of osteocalcin were achieved (*p* = 0.80 and *p* > 0.99, respectively) (Fig. 3a–d).

### The effect of testosterone or OMT on urinary DPD, Ca, and P

Urinary DPD of the SHAM group was insignificant compared to the CTRL group (*p* > 0.99). The urinary DPD and Ca were profoundly elevated in the ORX group when compared to the CTRL group (p < 0.001) and significantly declined following testosterone or OMT treatment (p < 0.001). OMT treatment was as effective as testosterone treatment in lowering urinary DPD levels and Ca levels (*p* > 0.99). In contrast, DPD values in testosterone- or OMT-treated rats were distinctively raised compared to the CTRL rats (p < 0.001). Changes in urinary Ca levels in the testosterone- or OMT-treated groups were insignificant compared to the CTRL group (*p* = 0.61 and *p* = 0.34, respectively) (Figs. 3e, 3f). Furthermore, variations in urinary P levels were of no statistical significance among all the study groups (*p* = 0.24) (Fig. 3g).

### The effect of testosterone or OMT on bone oxidant/antioxidant balance

**Fig. 4:**
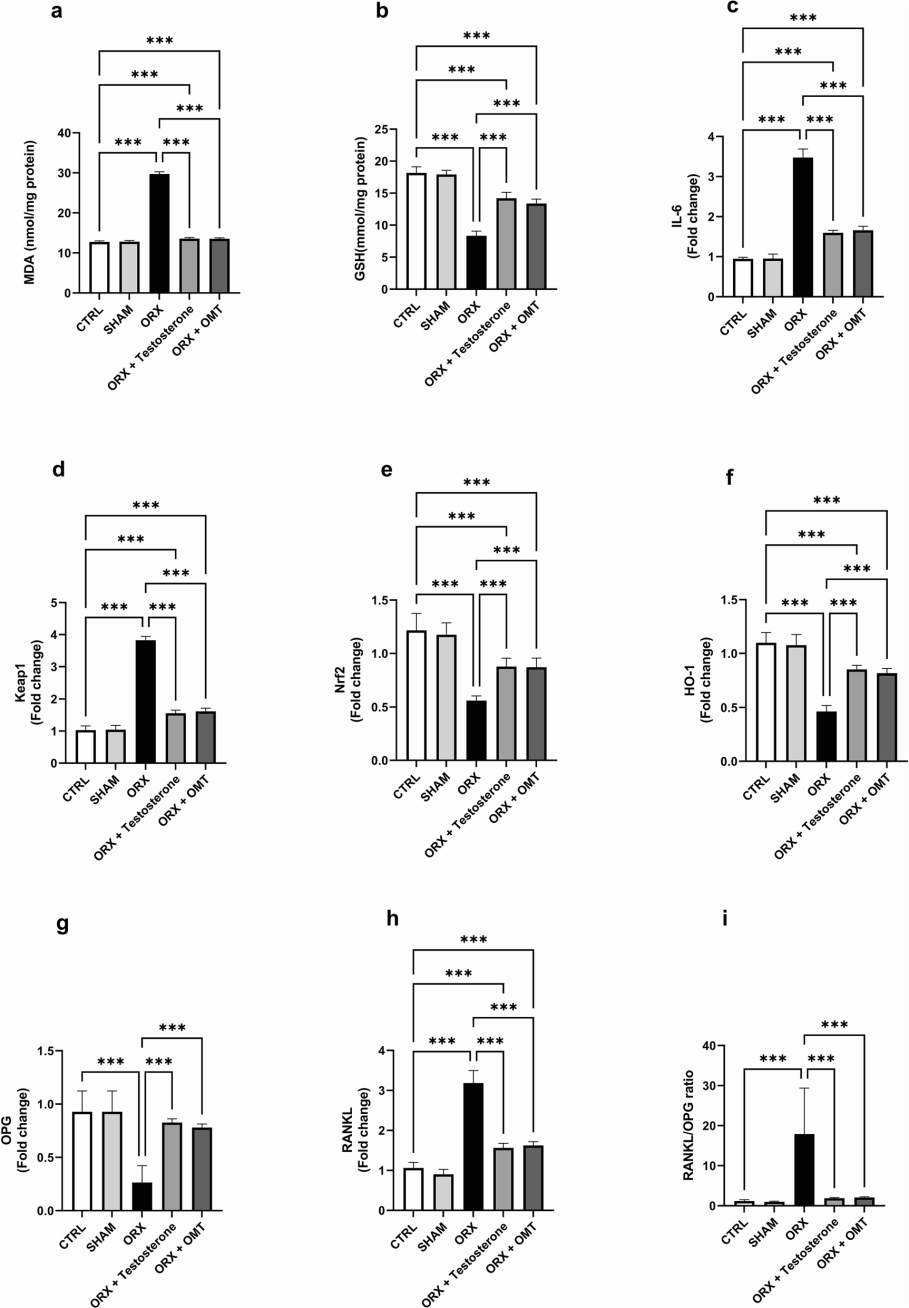
Testosterone or OMT ameliorated bone oxidant/antioxidant balance, inflammation, and RANKL/OPG expression ratio in ORX rats. **a** Bone MDA; **b** bone GSH; **c** IL-6 expression; **d** Keap1 expression; **e** Nrf2 expression; **f** HO-1 expression; **g** OPG expression; **h** RANKL expression; **i** RANKL/OPG ratio. Bar graphs were presented as mean ± SD (n = 12). Data analysis was conducted using one-way ANOVA and Bonferroni’s test for multiple comparisons. ^***^p < 0.001. ORX, orchiectomized; OMT, oxymatrine; MDA, malondialdehyde; GSH, reduced glutathione; IL-6, interleukin 6; Keap1, Kelch-like ECH-associated protein 1; Nrf2, nuclear factor erythroid 2-related factor 2; HO-1, heme oxygenase 1; OPG, osteoprotegerin; RANKL, receptor activator of nuclear factor κB ligand

Oxidant/antioxidant balance was detected by measuring bone MDA and GSH. Bone MDA and GSH levels in the SHAM group did not differ from the CTRL group values (*p* > 0.99). The ORX rats exhibited a marked rise in the bone MDA and a decline in bone GSH compared to the CTRL group (*p* < 0.001). Bone MDA and GSH levels were significantly restored in the testosterone- and OMT-treated rats. OMT treatment demonstrated the same efficacy as testosterone treatment in restoring the bone MDA and GSH levels (*p* > 0.99) while failing to achieve the values in the CTRL group (*p* < 0.001) (Fig. 4a–b).

### Testosterone or OMT affects bone IL-6, Keap1, Nrf2, HO-1, OPG, RANKL, and RANKL/OPG expression ratio

The bone expressions of IL-6, Keap1, HO-1, Nrf2, OPG, RANKL, and the RANKL/OPG ratio in the SHAM group closely resembled those of the CTRL group (*p* > 0.99). In comparison, the ORX group exhibited decreased Nrf2, HO-1, and OPG expressions (*p* < 0.001) in contrast to the CTRL group, while showing elevated IL-6, Keap1, RANKL levels and RANKL/OPG ratio (*p* < 0.001). After the testosterone or OMT treatment was applied, Nrf2, HO-1, and OPG levels were considerably improved, whereas IL-6, Keap1, RANKL, and the RANKL/OPG ratio declined compared to the untreated ORX group (*p* < 0.001). Compared to testosterone treatment, OMT treatment showed the same effect on the IL-6, Keap1, Nrf2, HO-1, OPG, RANKL, and RANKL/OPG expression ratio (*p* > 0.99). Furthermore, when comparing testosterone or OMT-treated animals to the CTRL ones, we found that Nrf2 and HO-1 were significantly reduced, while IL-6, Keap1, and RANKL were significantly elevated (*p* < 0.001). In contrast, OPG (*p* = 0.63 and *p* = 0.1, respectively) and RANKL/OPG ratio (*p* > 0.99) showed negligible differences (Fig. 4c–i, respectively).

### The effect of testosterone or OMT on osteocyte number and cortical thickness

**Fig. 5:**
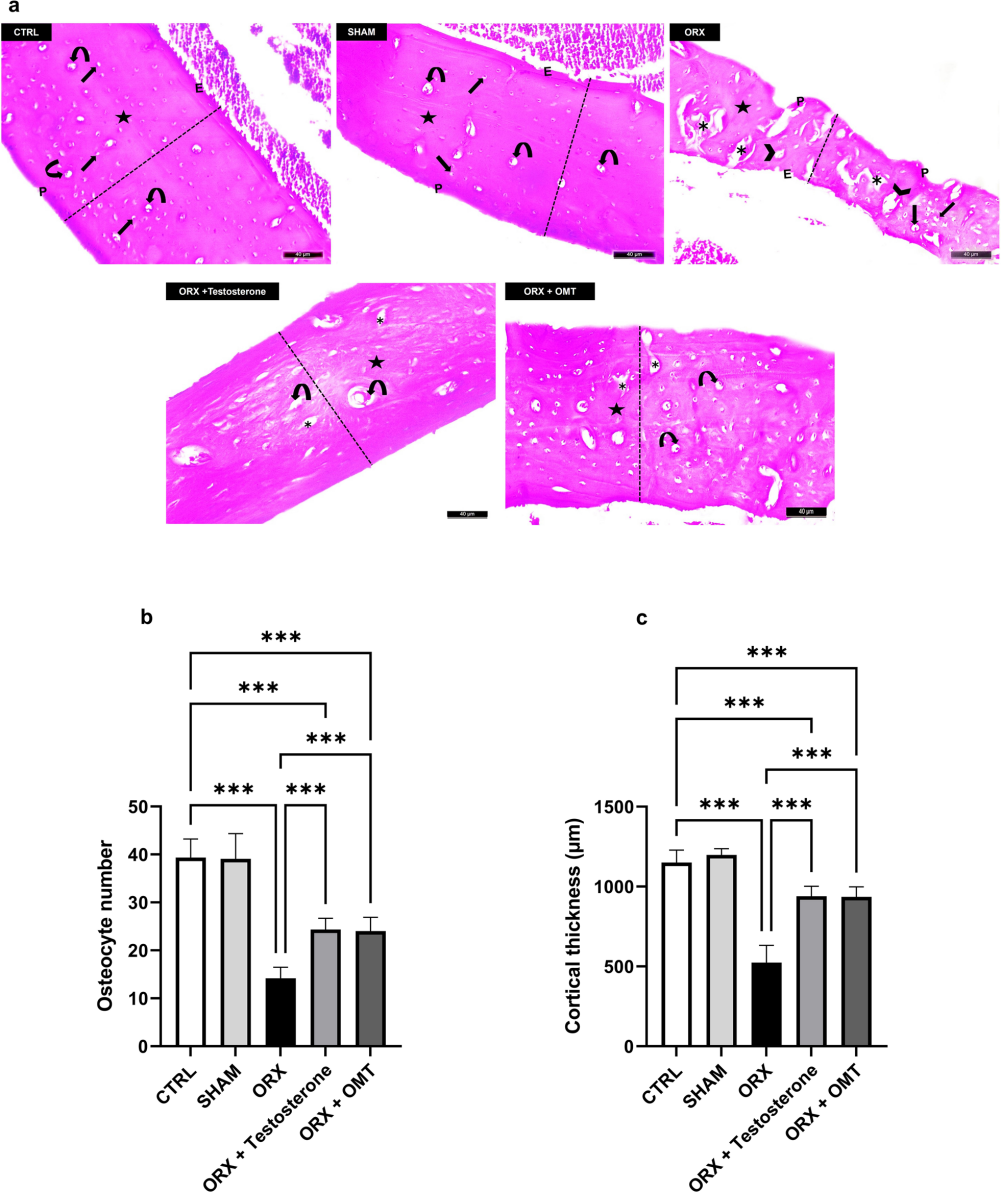
Testosterone or OMT ameliorated osteocyte number and cortical bone thickness in ORX rats. **a** Representative H&E-stained sections of rat femoral shaft: the CTRL and SHAM groups: revealing regular periosteum (P) and endosteum (E), regularly arranged bone lamellae with homogenous eosinophilic matrix (star). A number of osteocytes (arrows) were detected within their lacunae around well-organized Haversian canals (curved arrows). Normal cortical thickness of the femoral shaft was noticed (dashed line). The ORX group: showing separated irregular periosteum (P) and endosteum (E) and heterogenous bone matrix (star). Multiple osteoporotic cavities (asterisk), a few osteocytes with wide lacunae (arrows), and distortion of Haversian canals (arrowheads) are also observed. The cortical bone thickness (dashed line) was markedly reduced. The ORX + testosterone group: displayed apparently normal bone architecture with increased cortical thickness (dashed line). A homogenous bone matrix (star) was noticed in most zones. However, a few small bone cavities (asterisk) and distorted Haversian canals (curved arrows) were still observed. The ORX + OMT group: shows preservation of normal bone architecture with an almost homogenous bone matrix (star) and an apparent increase in cortical thickness (dashed line). Few bone cavities (asterisk) and wide osteocyte lacunae (curved arrows) are still observed. Scale bar = 40 µm, × 200; **b** Mean score of osteocyte number; **c** The mean score of cortical bone thickness. Bar graphs were presented as mean ± SD (n = 12). Data analysis was conducted using one-way ANOVA and Bonferroni’s test for multiple comparisons. ***p < 0.001. ORX, orchiectomized; OMT, oxymatrine

H&E-stained sections of rat femora of the CTRL and SHAM groups revealed regular periosteum and endosteum, as well as regularly arranged bone lamellae with a homogenous eosinophilic matrix. Many osteocytes were observed within their lacunae around well-organized Haversian canals. Also, a standard cortical thickness of the femoral shaft was noticed. The ORX group demonstrated separated irregular periosteum and endosteum and heterogenous bone matrix. Multiple osteoporotic cavities, a few osteocytes with wide lacunae, and distortion of Haversian canals were also detected. The cortical bone thickness was markedly reduced. The ORX + testosterone group displayed typical bone architecture with an increase in cortical thickness. A homogenous bone matrix was noticed in most zones. However, a few small bone cavities and a few distorted Haversian canals were still evident. The ORX + OMT group preserved the normal architecture of bone with a nearly homogenous bone matrix and exhibited a noticeable increase in cortical thickness. Moreover, few bone cavities and a few wide osteocyte lacunae were observed (Fig. 5a). The mean score of osteocyte number and cortical bone thickness in the SHAM rats was approximate as the CTRL rats’ values (*p* > 0.99, *p* = 0. 81, respectively). Orchiectomy resulted in the deterioration of osteocyte number and cortical bone thickness mean scores compared to normal rats (*p* < 0.001). Treatment with either testosterone or OMT showed almost equal therapeutic efficacy (*p* > 0.99) but resulted in lower osteocyte number and cortical bone thickness than those in the CTRL group (*p* < 0.001) (Figs. 5b and c).

### The effect of testosterone or OMT on bone TNF-α expression

**Fig. 6:**
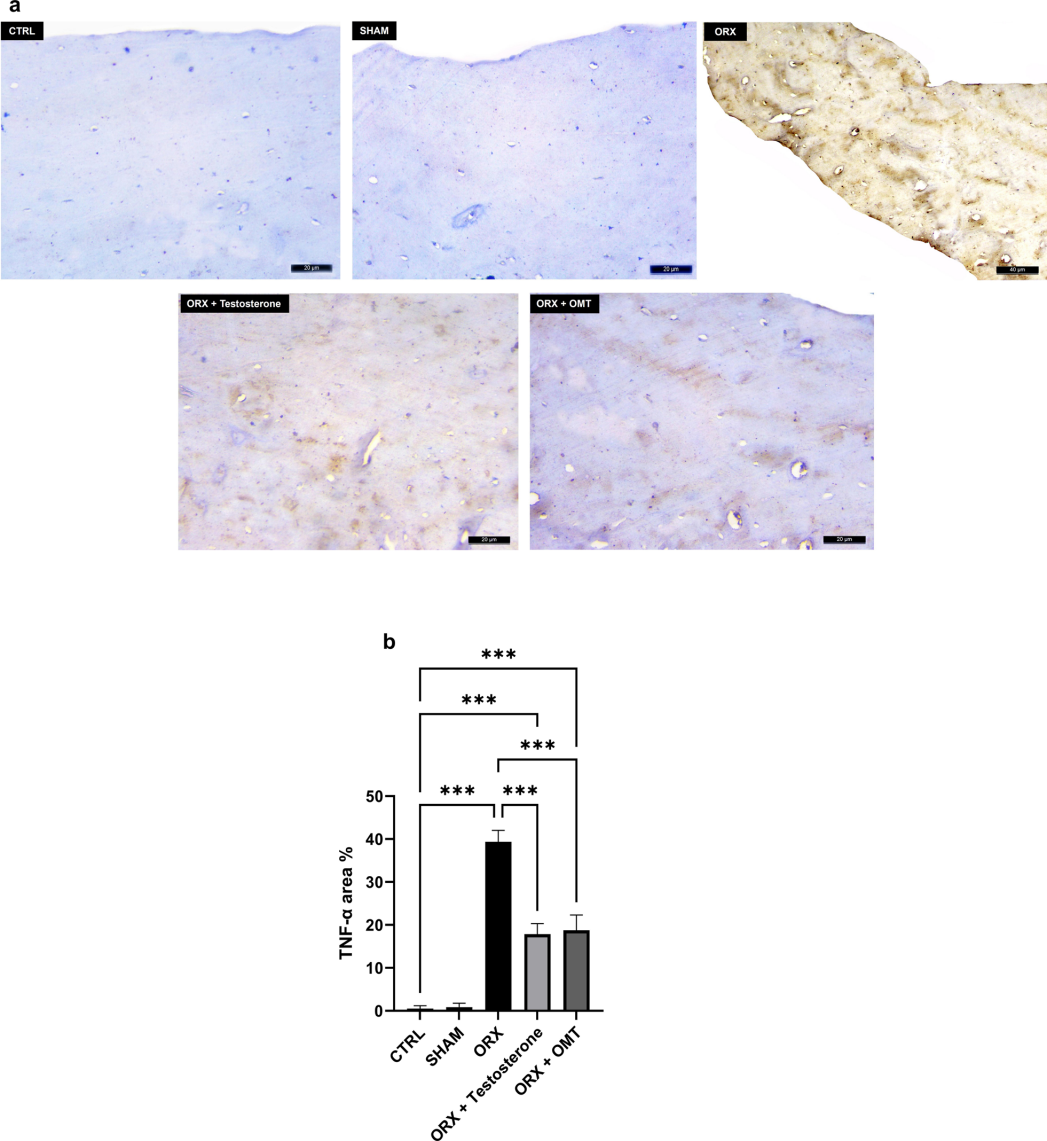
Representative TNF-α expression of rats’ femora. **a** The CTRL and SHAM groups displayed negative expressions. The ORX group demonstrated intense expression, while the ORX + testosterone and the ORX + OMT groups displayed moderate expression in the bone tissue. Scale bar = 20μm × 400; **b** Mean score of TNF-α area % bone expression. Bar graphs were presented as mean ± SD (n = 12). Data analysis was conducted using one-way ANOVA and Bonferroni’s test for multiple comparisons. ***p < 0.001. ORX, orchiectomized; OMT, oxymatrine; TNF-α, tumor necrosis factor-α

The values of bone TNF-α expression in the SHAM rats did not differ from the CTRL rats’ values (*p* > 0.99). In contrast, the ORX rats exhibited elevated TNF-α expression values than CTRL rats (*p* < 0.001), which were mitigated upon testosterone or OMT treatment (*p* < 0.001). Treatment with either testosterone or OMT showed the same efficacy in restoring the TNF-α expression levels (*p* > 0.99). However, both therapies led to higher TNF-α expression levels than those in CTRL (*p* < 0.001) (Fig. 6).

## Discussion

The ORX model in rats is an established experimental method for simulating male osteoporosis. Osteoporosis results from low testosterone levels due to orchiectomy, which decreases the formation of new bone [32]. Testosterone levels affect bone metabolism, both directly and indirectly; all bone-related cells and bone metabolism are directly impacted by testosterone [33].

The DEXA scan is an esteemed screening tool for the diagnosis and treatment of osteoporosis in most medical practices. It measures BMD and BMC, which are crucial for determining the strength, fragility, and risk of fracture of bone [34]. In this study, BMD was reduced in ORX rats compared to CTRL rats. The reduction of BMD was linked to a decrease in mineralization of the bone, as evidenced by a significant reduction in BMC. Furthermore, the number of osteocytes and cortical bone thickness was reduced in these rats with multiple osteoporotic cavities, wide lacunae, and distortion of Haversian canals as supported by previous studies [35–37]. The osteocytes’ supposed production of matrix components like collagen may be hindered by the decline in osteocyte count [38]. The observed decrease in BMD and BMC in ORX rats is consistent with the findings of Fekete et al. [39], Böker et al. [14], and Karesova et al. [40]. Treatment with testosterone or OMT ameliorated the reduced BMD and BMC and increased the number of osteocytes and cortical bone thickness in ORX rats. Böker et al. [14] revealed that testosterone therapy improved BMD and bone healing in ORX rats. Additionally, testosterone positively impacted bone structure as it increased trabecular bone formation by directly stimulating androgen receptors on osteocytes and osteoblasts. Also, continued supraphysiological administration of testosterone may improve mechanical strength, cortical thickness, and cortical area of bone [41, 42]. These observations support our findings. Testosterone suppressed osteoblast apoptosis and triggered osteoblast differentiation [32]. Furthermore, OMT effectively ameliorated ovariectomy-induced osteopenia by obstructing osteoclastogenesis in vivo [43, 44].

To support our results for BMD and BMC, we measured the markers of bone turnover. Recently, several biomarkers gained popularity for assessing bone turnover rate and monitoring osteoporosis treatment response [45–47]. Therefore, in the present study, we analyzed serum for osteocalcin, ALP, NTXI, and TRAP levels and urine for DPD. These biomarkers can yield valuable insights into the bone remodeling processes [14]. In the current investigation, elevated serum osteocalcin levels and ALP indicated that the ORX rats had high turnover osteoporosis. Similar findings were reported previously [33, 48, 49]. Osteocalcin is the second most prevalent protein in the bone matrix and is responsible for bone mineralization and hydroxyapatite crystal formation. It was considered a specific indicator of osteoblast activity and the rate of formation of bone. Nonetheless, bone matrix-embedded osteocalcin is also liberated during bone resorption, and osteoporosis results in increased serum osteocalcin levels [50, 51].

ALP is a byproduct produced during the bone remodeling process. Serum ALP serves as an essential indicator for assessing osteoporosis [52]. We observed increased levels of TRAP and NTXI in serum and levels of DPD in urine in ORX rats compared to the CTRL group, which was consistent with earlier studies [6, 53].

Osteoclasts normally secrete TRAP in bone resorption. TRAP is a widely used osteoclast marker that characterizes the activity and number of osteoclasts. Additionally, DPD and NTXI are produced due to the osteoclasts’ bone matrix degradation process. They are also sensitive markers for assessing the risk of fracture in postmenopausal women [54, 55]. We observed an increase in these bone turnover biomarkers in ORX rats, which was reversed in testosterone or OMT-treated ORX rats. Consistent with our findings, other researchers found that testosterone decreased bone remodeling and reduced biochemical markers of bone turnover [41, 56].

The current investigation revealed that reduced serum testosterone level in ORX rats was accompanied by a considerable increase in serum PTH, a significant decrease in serum Ca level, and an insignificant change in serum P that was associated with a substantial increase in urinary Ca excretion and negligible change in urinary P. These outcomes were significantly reversed in individual treatment with testosterone or OMT in ORX rats. In osteoporosis, Ca and P levels are generally deficient. The drop in Ca reabsorption following an orchiectomy contributes to osteoporosis, and young males with hypogonadism experience a notable increase in Ca losses in urine, with bone Ca accounting for the majority of these losses [57, 58]. Intestinal Ca malabsorption may also lead to hypogonadism-induced osteoporosis, causing a feedback rise in the PTH that promotes bone resorption and maintains the blood’s Ca levels balanced [59]. These results are in line with those of prior studies [60, 61]. Furthermore, testosterone enhances renal Ca reabsorption [62]. Serum Ca level was significantly elevated which was accompanied by a significant decrease in urinary Ca, and serum PTH was reduced considerably in OMT-treated ORX rats compared to CTRL rats in the present study. The amelioration of oxidative stress can explain this, as it was reported that exacerbation of ROS production results in oxidative stress, which inhibits intestinal Ca absorption [63].

The bone is a dynamic structure continuously undergoing formation and breakdown regulated by bone-resorbing osteoclastic and bone-synthesizing osteoblastic cells. Several signaling pathways can regulate the process of osteoclast maturation and bone remodeling, including RANKL/OPG. The potential link between RANKL, OPG, and BMD levels has been studied [64]. RANKL is a final downstream protein cytokine secreted by osteoblasts and osteocytes. It is produced both as a free-secreted protein and as a membrane protein. It activates the RANK transmembrane receptor expressed by osteoclasts and its precursors, leading to their activation and maintenance. RANKL’s biological activity is modulated by its physiological decoy receptor, OPG. OPG has a solid affinity to RANKL and prevents RANKL-RANK association, thus inhibiting osteoclastogenesis. In this study, the RANKL/OPG ratio was substantially increased within ORX rats, indicative of amplified osteoclast activity and subsequent bone resorption [65]. These findings align with those in previous research by Beranger et al. and Komrakova et al. [66, 67]. While elevated plasma OPG levels are typically correlated with increased bone mass in postmenopausal women, a significant reduction in the RANKL/OPG ratio following testosterone treatment in ORX rats was demonstrated here. Previous work by Chen et al. [68] illustrated that testosterone upregulates OPG mRNA expression in murine bone cell cultures and osteoblastic cell lines, likely through androgen receptor mediation. Furthermore, testosterone can be transformed into estradiol via aromatase enzymes, which have been shown to suppress RANKL expression within bone marrow cells [44].

The RANKL expression is significantly induced and influenced by many osteoactive agents, including glucocorticoids, vitamin D3, IL-1, and TNF-α [69, 70]. Additionally, the critical osteoclast differentiation-inducing molecule RANKL and its decoy receptor OPG can be directly regulated by sex steroids [71] and PTH hormone [72].

Numerous research studies indicated that inflammation and oxidative stress play pivotal roles in the progression of osteoporosis. These elements impede osteoblast differentiation, foster osteoclasts’ differentiation and activity, elevate apoptotic osteocytes, and increase the RANKL/OPG ratio and RANKL expression levels. Oxidative stress is a robust inducer for the expression of pro-resorptive cytokines such as IL-1, TNF-α, and IL-6, culminating in osteoporosis [9]. The potent pro-inflammatory cytokine TNF-α stimulates osteoclast formation and bone loss. TNF-α amplifies RANKL-induced osteoclastogenesis by activating the NF-κB and PI3K/Akt signaling pathways [73]. A marked increase in serum MDA levels and a notable decrease in serum GSH levels were detected in the ORX rats. Additionally, a noteworthy increase in TNF-α immunoreactivity and IL-6 was observed in the bone of ORX rats compared to the CTRL group. The results were consistent with previous studies’ findings [74]. Numerous animal models have been used to study the correlation between elevated inflammatory biomarkers and androgen deficiency. Bianchi demonstrated a correlation between testosterone deficiency and higher expression of inflammatory markers such as TNF-α. Furthermore, orchidectomy induced a significant increase in IL-6 in male rats [75, 76]. Serum levels of IL-6 and BMD had a significant negative correlation. The authors noted that IL-6 negatively regulates osteoblast differentiation and contributes to defective osteogenesis [77].

Low levels of testosterone are associated with oxidative stress [78]. Nrf2 is a transcription factor that controls cellular defense mechanisms against oxidative stress and inflammation by regulating the expression of antioxidant responses element–dependent genes, affecting physiological and pathophysiological outcomes of oxidant exposure. Reduction in the Nrf2 signaling system’s adaptive response has dramatically influenced the accumulation of oxidative damage [79]. Downregulation of Nrf2 was observed in a rat model of late-onset hypogonadism [80]. We have observed that the Nrf2 levels in ORX rats were lower than those in the CTRL group. Nrf2 is extensively expressed in osteoblasts, osteoclasts, and other bone cells. Its activation induces the expression of various antioxidant and detoxification enzymes, such as HO-1. HO-1 level was downregulated in the present study. HO-1 is a stress-response protein that plays a vital role in the cellular adaptive and/or protective response to oxidative stress and inflammation, as well as the maintenance of cellular homeostasis [81]. Research has demonstrated that a loss in Nrf2/HO-1 deteriorated bone density during bone remodeling, increased the production of RANK ligands, and encouraged bone resorption through osteoclastogenesis [82]. Moreover, numerous studies suggested that increased production of Nrf2 in the nucleus helps reduce the imbalance of bone remodeling, improving the healing of fractures, lowering the risk of osteoarthritis, and increasing resistance to tumors [83]. A significant increase in bone keap1 level was observed in ORX rats. Keap1 is a repressor protein that binds to Nrf2 and enhances its degradation by the ubiquitin–proteasome pathway. Under physiological conditions, Nrf2 is sequestered in the cytoplasm by Keap1. Under oxidative stress, the Keap1-Nrf2 complex is resolved causing Nrf2 to translocate into the nucleus and express the target genes [84, 85].

In the current study, individual treatment with testosterone or OMT in ORX rats significantly reduced serum levels of MDA and bone levels of Keap1, TNF-α, and IL-6 while considerably elevating serum GSH and bone Nrf2 and HO-1 levels. Consistent with our results, testosterone might exhibit anti-inflammatory and antioxidative characteristics. Testosterone replacement therapy decreased the pro-inflammatory cytokine TNF-α and mitigated oxidative stress by lowering MDA levels and enhancing GSH levels [86–88]. Furthermore, testosterone activated the Nrf2/HO-1 antioxidant response element pathway in aging rats [89].

In several research endeavors, OMT has demonstrated a spectrum of advantageous properties, encompassing antioxidative, anti-inflammatory, antiviral, and anticancer attributes, along with immune system modulation, enhancement of cardiovascular performance, and safeguarding of the nervous system [13, 90, 91]. The current literature on the antiosteoporotic effects of OMT is limited. However, our research has uncovered a potential breakthrough. Notably, OMT treatment significantly improved BMD and BMC in ORX rats, preserving typical bone architecture and increasing osteocyte number and cortical thickness. Furthermore, bone turnover markers were also improved considerably. These positive outcomes, which may be linked to OMT’s anti-inflammatory and antioxidative properties, offer a ray of hope for future research and potential clinical applications. In this study, OMT ameliorated the elevated bone IL-6 and TNF-α. It also reduced the generation of other inflammatory mediators such as prostaglandin E2 [92]. OMT exerts anti-inflammatory properties by downregulating NF-kB-induced inflammatory response [93]. In contrast, OMT ameliorated oxidative stress and enhanced the expression of Nrf2/HO-1 in ORX rats. It also reduced the progression of osteoarthritis and ameliorated myocardial injury by activating Nrf2 and inhibiting oxidative stress and apoptosis in rat models of osteoarthritis and type 2 diabetes [94, 95]. The antioxidant properties of OMT may involve the activation of sirtuin 1 (SIRT1)/Nrf2 signaling that promotes the capacity of antioxidant enzymes to scavenge oxygen free radicals [96] or by activating AMP-activated protein kinase [97]. We also observed increased Nrf2 and OPG expression and decreased Keap1, RANKL, and RANKL/OPG ratio in the OMT-treated ORX group. Testosterone replacement has shown promise in treating hypogonadism and enhancing sexual performance, bone density, muscle strength, mood, and cognitive abilities. However, physicians ought to be aware of the drawbacks. The following common side effects were reported: erythrocytosis, venous thromboembolism, male infertility, testicular atrophy, gynecomastia, and growth of prostate cancer. Furthermore, the relationship between exogenous testosterone treatment and cardiovascular risks, such as the risk of heart attack and stroke, was issued. Therefore, physicians seek medical and natural alternatives to life-long testosterone therapy [98].

## Conclusion

According to the above discussion, we concluded that individual treatment with testosterone or OMT ameliorated osteoporosis in ORX rats that was associated with alleviated inflammatory cytokines, TNF-α and IL-6, reduced MDA and Keap1, enhanced GSH, and upregulated Nrf2/HO-1 with observed non-significant changes between both treatment groups. Nevertheless, OMT mechanisms are varied and intricate, and they require further research and investigation.

## Data Availability

No datasets were generated or analysed during the current study.
